# Psychometric Evaluation of the Chinese Version of Occupational LowBack Pain Prevention Behaviors Questionnaire Among Clinical Nurses: A Validation Study

**DOI:** 10.3389/fpubh.2022.827604

**Published:** 2022-03-23

**Authors:** Chunqi Zhang, Zhen Yang, Huijun Zhang

**Affiliations:** ^1^Department of Nursing, The First Affiliated Hospital of Jinzhou Medical University, Jinzhou, China; ^2^Department of Nursing, Jinzhou Medical University, Jinzhou, China

**Keywords:** health promotion, occupational low back pain, clinical nurses, factor analysis, psychometric evaluation

## Abstract

**Objective:**

This study aimed to translate and validate of the Chinese version of the Occupational Low Back Pain Prevention Behaviors Questionnaire among clinical nurses.

**Methods:**

A total of 1,186 clinical nurses were recruited from three provinces in northeast China. The reliability of the translated questionnaire was measured by internal consistency, split-half reliability, and test-retest reliability. The validity of the translated questionnaire was evaluated by content validity index, exploratory factor analysis and confirmatory factor analysis.

**Results:**

The Cronbach's α value of the questionnaire was 0.891, and the coefficient values for the six domains ranged between 0.804 and 0.917. The split-half reliability and test-retest reliability were 0.663 and 0.734, respectively. Furthermore, the content validity index of the questionnaire was 0.938. The 6-factor structure, supported by the eigenvalues, total variance explained, and scree plot accounted for 63.038% of the total variance. In the confirmatory factor analysis, as the results of model fitting, χ^2^/df = 3.753, RMSEA = 0.048, GFI = 0.929, AGFI = 0.913, TLI = 0.934, IFI = 0.943, CFI = 0.943, PGFI = 0.759, PNFI = 0.807.

**Conclusion:**

The Chinese version of the Occupational Low Back Pain Prevention Behaviors Questionnaire had suitable reliability and validity among clinical nurses. Under the high prevalence of occupational low back pain, the questionnaire can provide a reference for developing educational intervention plans among clinical nurses.

## Introduction

Occupational low back pain is one of the most common work-related health problems among health care professionals, especially clinical nurses. The prevalence of occupational low back pain in nurses is about 50.0 to 84.7% in European countries and 86.1% in China (1–3). It was reported that 77% of nurses will have definite symptoms of low back pain after 1 year on the job (4). The empirical study showed that the absence rate caused by occupational low back pain among nurses was about 27.8% (5), and the turnover rate was as high as 58% (6), which seriously affects the quality of clinical nursing and the efficiency of nursing work. How to effectively prevent occupational low back pain has become an urgent problem among nurses.

Occupational low back pain is a kind of occupational health problem that causes related symptoms due to the long-term involvement of the low back under the influence of occupational factors (7). As the vulnerable group, the prevalence of occupational low back pain among nurses is four times higher than that in other healthcare professionals (8). Age, longtime standing, poor sitting habits, heavy workload and improper working posture are the main factors of occupational low back pain prevalence among nurses (9, 10). Moreover, nurses in understaffed departments were 2.74 times more likely to develop chronic low back pain than those in adequately staffed departments (11). It was also reported that more than half of nurses suffer from low back pain because of insufficient training in relevant knowledge and protective skills (12, 13). In addition, psychosocial factors were also reported to be closely associated with nurses' occupational low back pain (14). It mainly include negative attitude, fear-avoidance behavior, negative expectation, depressive tendency and social avoidance of low back pain (15–17). The existence of these factors suggested the importance of evaluating the psychosocial status of nurses with low back pain.

Occupational low back pain will not only make nurses have negative emotions and reduce the quality of life, but also lead to the decline of their working ability and the quality of professional life, and then reduce the quality of nursing, threatening the health and safety of patients (13, 18). Occupational low back pain among nurses imposed a huge medical and economic burden on individuals, families, communities and governments (19). According to the research data of the British Health and Safety Executive in 2017, the absenteeism caused by occupational low back pain reaches 2.2 million working days (20), which seriously leads to the shortage of human resources and is not conducive to the stability of the healthcare system. More dangerously, the occupational low back pain may be exacerbated by the long course of disease and the complexity of the job (21). At the same time, the lack of knowledge about the prevention of occupational low back pain will reduce the health promotion beliefs and behaviors of clinical nurses and greatly increase the risk of lifelong disability (22).

As a health promotion behavior, prevention plays an irreplaceable role in the healthcare field. There are measurement tools that can accurately assess pain intensity, pain-related disability, and low back pain disability (22, 23). However, these tools do not examine the effective factors that might help to promote health behaviors. Therefore, from the salient features of health promotion and arising out of the PRECEDE-PROCEED model, the occupational low back pain prevention behaviors questionnaire was developed (24). The prevention of occupational low back pain is a repeated process of assessing prevention knowledge and the practice of prevention behavior to meet the professional's internal and external needs in work (25). The development of the occupational low back pain prevention behaviors questionnaire has arisen out of this need to assess participants' level of prevention behaviors in their daily practice. Despite the highlighted benefits of the prevention of occupational low back pain in the physiology and psychology of the individual, but research on the prevention of occupational low back pain is still incomplete in China, especially lacking a tool to evaluate the occupational low back pain prevention behaviors among nurses.

Therefore, the purpose of this study was to translate the occupational low back pain prevention behavior questionnaire into Chinese and validated its reliability and validity. We put forward the hypothesis that the Chinese version of occupational low back pain prevention behavior questionnaire has satisfactory psychometric properties.

## Methods

### Design and Participants

In this study, a multi-center cross-sectional survey was conducted from March to September 2021. This study included three stages of instrument localization (26): (a) Translation and cross-cultural adaptation of the questionnaire; (b) Item analysis of the questionnaire; (c) Evaluation of psychometric properties of the questionnaire. In this study, in order to ensure the accuracy of the research results, 20 participants are required for each item. The questionnaire contains 30 items, and 600 participants should be recruited. However, 25% sampling error and 25% sample loss rate were also considered and a larger sample is desirable (27, 28). Therefore, we finally recruited 1,186 clinical nurses by convenient sampling in hospitals in northeast China. The inclusion criteria were registered nurses with more than 1 year of clinical experience. The exclusion criteria were nurses from Non-clinical departments and nurses who are not affiliated to this hospital (visiting nurses).

### Measures

#### Background Characteristics

Based on comprehensive literature review and careful group discussion, the general demographic characteristics questionnaire was designed. This questionnaire includes six self-report items, namely age, gender, education level, marital status, location and professional experience.

#### Occupational Low Back Pain Prevention Behaviors Questionnaire

The level of occupational low back pain prevention behaviors was measured by the occupational low back pain prevention behaviors questionnaire developed by Kazemi et al. (24). The questionnaire consisted of six dimensions with 30 items. The dimensions were named knowledge, attitude, self-efficacy, reinforcing factors, enabling factors, and behavior, respectively. The items on knowledge dimension came in the form of a question where the correct answers assigned 1 and wrong answers or “I do not know” assigned 0. In the items on other dimensions, the Likert 5-grade scoring system was used to collect participants' responses. The higher the total score, the higher the level of occupational low back pain prevention behaviors. The Cronbach's α value was 0.92, with domains ranging from 0.49 to 0.87.

### Procedure

#### Data Collection Procedure

**Figure 1:**
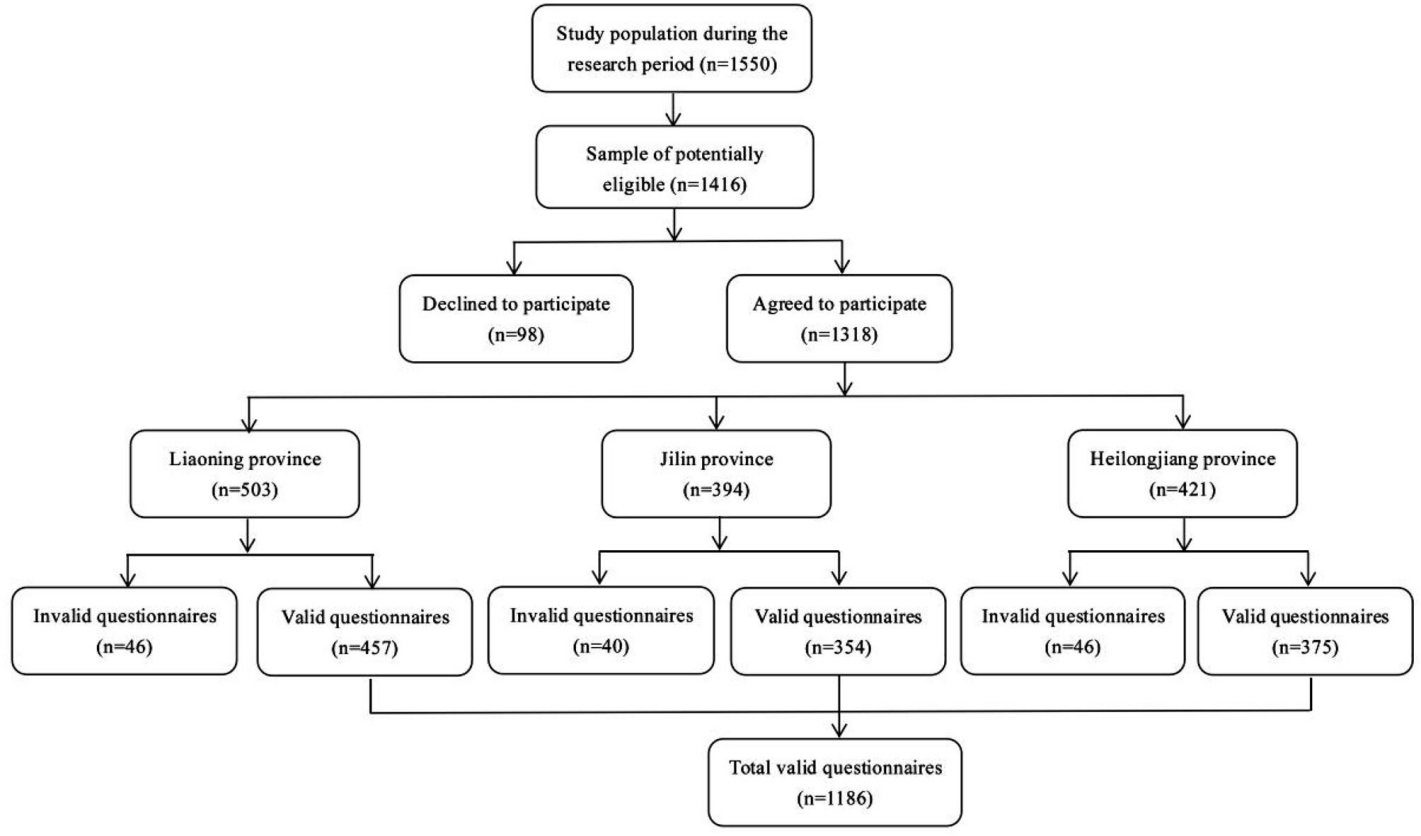
Flowchart of participants.

In this study, investigators went to three cities in northeast China to recruit participants by using convenience sampling after unified training. Based on the previous investigation, we estimated that we could contact about 1,500 clinical nurses during the study period. During the formal investigation, 1,416 eligible clinical nurses were invited to participate in this study, among which 98 clinical nurses refused to participate in this investigation due to heavy clinical tasks. Participants were invited to the quiet classroom of the hospital to fill out the questionnaire anonymously. In the end, we excluded 132 invalid questionnaires and got 1,186 valid ones. The specific process is shown in Figure 1.

#### Questionnaire Translation Procedure

With the authorization of Professor Montazeri, Brislin's double literal translation-back translation model (29) was adopted to translate the questionnaire into Chinese, and the first draft was culturally adapted by Delphi expert consultation. In the literal translation stage, two professors majoring in English were invited to translate the original questionnaire, and then the researcher and professors discussed and negotiated the controversial points in the translation results to determine the literal translation version. In the back translation stage, two foreign English professors who are native English speakers and have not read the original scale in domestic medical colleges back translated the literal translation version, and the researcher discussed with professors to determine the back translation version. Finally, the researcher, together with four translators in literal translation stage and back translation stage, compared the back translation version with the original questionnaire, and discussed the ambiguities to determine the first draft of the Chinese version of occupational low back pain prevention behavior questionnaire. Seven experienced experts in health promotion and psychometrics were invited to revise the design and content of the questionnaire and make cultural adaptation (30).

#### Data Analysis Procedure

Data were analyzed using SPSS 22.0 and AMOS 23.0 statistical software (IBM Corporation, Armonk, NY). All statistical tests were 2-sided, and *P* < 0.05 was considered statistically significant.

##### Items Analysis

The total score of the questionnaire was calculated and ranked from high to low, and the clinical nurses with the top 27% (high-score group) and the bottom 27% (low-score group) of the total score were determined. The independent sample *t* test was used to assess the significance of the difference between the nurses with high-score group and low-score group. In addition, the item-questionnaire correlation and the Cronbach's α value if item deleted were analyzed to assess the discrimination. Our requirements were as follows: (1) the correlation coefficient of each item-questionnaire >0.4; (2) the Cronbach's α value after deleting the item does not exceed the original Cronbach's α value; and (3) the critical ratio (CR) of each item >3.0.

##### Reliability Analysis

The internal consistency of the translated questionnaire was evaluated by the Cronbach's α value, split-half reliability and retest reliability. Our requirements were as follows: (1) the Cronbach's α value of the questionnaire and its dimensions ≥0.7; (2) the split-half reliability coefficient ≥0.6; and (3) the retest reliability coefficient ≥0.7.

##### Validity Analysis

We invited seven experts in the field of health promotion to evaluate the content validity of the translated questionnaire. Based on the content, the Likert 4-grade scoring system was used to collect experts' responses (from irrelevant to highly relevant). Irrelevant and somewhat relevant were assigned to 0 point and relevant and high relevant were assigned to 1 point. The content validity index of the items (I-CVI) is the ratio of the number of experts who ranked each item with 1 point to the total number of experts. The content validity index of the questionnaire (Q-CVI) is the mean of I-CVI for all items. The I-CVI ≥ 0.7 and the Q-CVI ≥ 0.9 were desired. The potential factor structure of the translated questionnaire was evaluated by exploratory factor analysis (EFA) and confirmatory factor analysis (CFA). EFA was determined by the results of Kaiser-Meyer-Olkin (KMO) and Bartlett test of sphericity. Generally, it is considered that KMO > 0.6 and Bartlett spherical test difference reached significant level (*P* < 0.05) is the premise of EFA. The principal component analysis (PCA) was used to extract common factors, and the maximum variance rotation method was used to extract common factors with initial eigenvalues >1. We combined the scree plot with the rotated factor structure to comprehensively judge whether the common factors of the questionnaire are retained. Moreover, we also used the AMOS in CFA and evaluated the applicability of the model by fitting the index.

### Ethical Approval

Based on strict ethical requirements (31), we obtained informed consent signed by all participants and anonymous questionnaires. The 1964 Helsinki Declaration was strictly observed throughout our study (32). Our study protocol was approved by the Ethics Committee of the Jinzhou Medical University (JZMULL2021005).

## Results

### Descriptive Statistics

This study included 1,186 nurses: 289 males (24.4%) and 897 females (75.6%). 41.1% of participants were between 25 and 34 years old. 64.1% of participants was married. 44.3% of the participants had an undergraduate education. 41.6% of participants have been in clinical care for 6–10 years. 38.5% of participants came from Liaoning province (Table 1).

**Table 1:** Frequency distribution of demographic characteristics (*n* = 1,186).

**Factors**	**Group**	***n***	**%**
Age	18–24	357	30.1
	25–4	497	41.9
	35–44	283	23.9
	≥45	49	4.1
Sex	Male	298	24.4
	Female	897	75.6
Education level	Junior college education	387	32.6
	Undergraduate education	525	44.3
	Postgraduate education	274	23.1
Marital status	Unmarried	397	33.5
	Married	728	61.4
	Divorced/Widowed	61	5.1
Site	Liaoning province	457	38.5
	Jilin Province	354	29.9
	Heilongjiang province	375	31.6
Professional experience (year)	1–5	163	32.0
	6–10	212	41.6
	11–15	69	13.5
	16–20	43	8.4
	≥20	23	4.5

### Translation and Cross-Cultural Adaptation

Due to the differences in language and culture, the translated questionnaires need to be cross-cultural adapted. Given that knowledge dimension appear in the form of questions, and the Cronbach's α coefficient is low in the original author's report, it is modified into likert scale based on the cultural background of mainland China, which is consistent with the scoring standard of other items. The revised items include the following: I know what is occupational low back pain; I know the causes of occupational low back pain; I know what caused the lumbar injury; I know the treatment methods of occupational low back pain. The first draft of the Chinese version of the occupational low back pain prevention behaviors questionnaire was developed. Then, the Delphi method was used to invite 7 experts in nursing management, nursing education, psychological measurement, occupational health and other fields to conduct expert consultation. Finally, 30 items were retained to form the final Chinese version of the Occupational Low Back Pain Prevention Behaviors Questionnaire.

### Item Analysis

The CR of the translated questionnaire items with *t-*value <0.05 ranged from 8.122 to 24.480, all of which >3.000, indicating that all items of the translated questionnaire had a high discrimination. The correlation coefficient (r) of item-questionnaire ranged from 0.402 to 0.634 (*P* < 0.001), which was within the acceptable range. After deleting each item one by one, the total Cronbach's α coefficient of the translated questionnaire ranges from 0.885 to 0.890, which does not exceed the original Cronbach's α coefficient. Based on the above, the 30 items of the translated questionnaire are retained. The detailed data information was shown in Table 2.

**Table 2:** Item analysis for Chinese version of the occupational low back pain prevention behaviors questionnaire.

**Item**	**Critical ratio**	**Correlation coefficient between item and total score**	**Cronbach's Alpha if item deleted**
Knowledge-1	24.422	0.638	0.885
Knowledge-2	16.997	0.534	0.887
Knowledge-3	22.016	0.628	0.885
Knowledge-4	23.740	0.634	0.885
Attitude-1	20.714	0.528	0.887
Attitude-2	12.259	0.480	0.890
Attitude-3	19.886	0.535	0.887
Attitude-4	23.374	0.562	0.887
Attitude-5	16.380	0.440	0.889
Self-Efficacy-1	22.970	0.598	0.886
Self-Efficacy-2	17.359	0.496	0.888
Self-Efficacy-3	24.480	0.624	0.885
Self-Efficacy-4	20.809	0.555	0.887
Self-Efficacy-5	16.126	0.453	0.889
Self-Efficacy-6	14.848	0.435	0.890
Reinforcing-1	18.746	0.510	0.888
Reinforcing-2	20.449	0.504	0.888
Reinforcing-3	20.727	0.541	0.887
Reinforcing-4	20.115	0.528	0.887
Reinforcing-5	12.461	0.459	0.890
Enabling-1	18.273	0.483	0.888
Enabling-2	15.710	0.434	0.889
Enabling-3	19.294	0.505	0.888
Enabling-4	18.327	0.488	0.888
Enabling-5	8.122	0.402	0.890
Enabling-6	16.630	0.448	0.889
Enabling-7	19.290	0.498	0.888
Behavior-1	13.485	0.409	0.890
Behavior-2	10.513	0.422	0.889
Behavior-3	13.313	0.485	0.890

### Reliability Analysis

The Cronbach's α value of Chinese version of the questionnaire was 0.891, and the Cronbach's α value of dimension was 0.804 0.917. Furthermore, based on the parity of the item number of the questionnaire, it was divided into odd and even groups. The correlation coefficient of the scores of the two groups was the split-half reliability coefficient, and the split-half reliability coefficient of the Chinese version of the questionnaire was 0.663. Two weeks later, we selected 80 nurses to retest, and the test-retest reliability coefficient of Chinese version of the questionnaire was 0.734.

### Validity Analysis

#### Content Validity Analysis

In this study, seven experts in the field of health promotion were invited to evaluate the content validity of the translated questionnaire. I-CVI and Q-CVI were used as important indexes to evaluate the content validity. The results showed that the I-CVI ranged from 0.875 to 1.000, and the Q-CVI was 0.938.

#### Exploratory Factor Analysis

**Figure 2:**
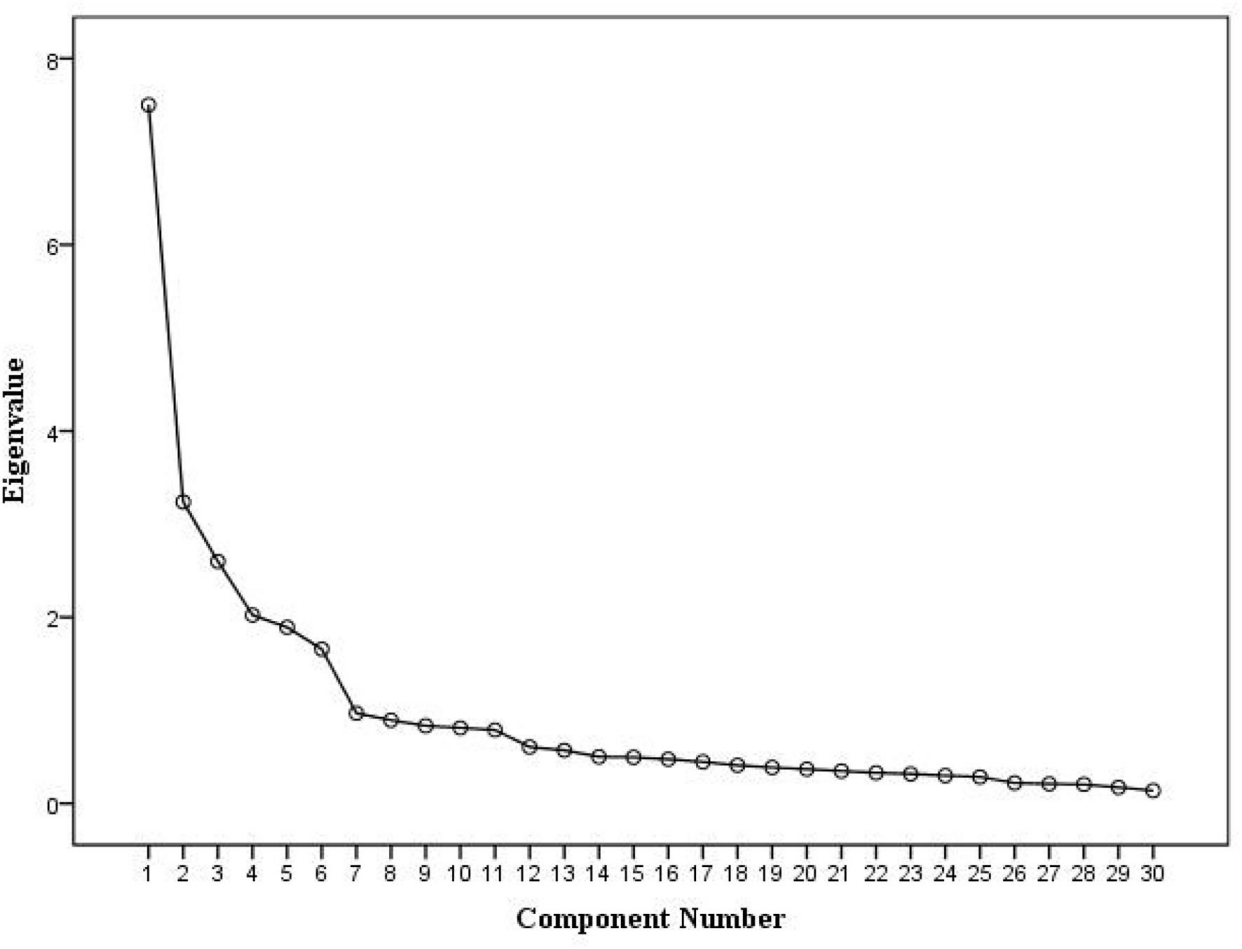
Screen plot of exploratory factor analysis for Chinese version of the occupational low back pain prevention behaviors questionnaire.

The KMO value of the translated questionnaire was 0.860, and the chi-square value of the Bartlett test of sphericity was 18530.540 (*P* < 0.05), which was suitable for factor analysis. PCA was used to extract six common factors whose initial eigenvalues >1, which was further confirmed by the scree plot (Figure 2), and there was no multiple factor loadings (Table 3). Six factors explained 63.038% of the total variance.

**Table 3:** Factor loadings of exploratory factor analysis for Chinese version of the occupational low back pain prevention behaviors questionnaire.

**Item**	**Factor 1**	**Factor 2**	**Factor 3**	**Factor 4**	**Factor 5**	**Factor 6**
Knowledge-1	-	-	-	-	0.843	-
Knowledge-2	-	-	-	-	0.826	-
Knowledge-3	-	-	-	-	0.836	-
Knowledge-4	-	-	-	-	0.858	-
Attitude-1	-	-	0.736	-	-	-
Attitude-2	-	-	0.762	-	-	-
Attitude-3	-	-	0.719	-	-	-
Attitude-4	-	-	0.768	-	-	-
Attitude-5	-	-	0.798	-	-	-
Self-Efficacy-1	-	0.812	-	-	-	-
Self-Efficacy-2	-	0.703	-	-	-	-
Self-Efficacy-3	-	0.794	-	-	-	-
Self-Efficacy-4	-	0.610	-	-	-	-
Self-Efficacy-5	-	0.714	-	-	-	-
Self-Efficacy-6	-	0.679	-	-	-	-
Reinforcing-1	-	-	-	0.795	-	-
Reinforcing-2	-	-	-	0.778	-	-
Reinforcing-3	-	-	-	0.793	-	-
Reinforcing-4	-	-	-	0.807	-	-
Reinforcing-5	-	-	-	0.528	-	-
Enabling-1	0.739	-	-	-	-	-
Enabling-2	0.730	-	-	-	-	-
Enabling-3	0.790	-	-	-	-	-
Enabling-4	0.727	-	-	-	-	-
Enabling-5	0.508	-	-	-	-	-
Enabling-6	0.732	-	-	-	-	-
Enabling-7	0.804	-	-	-	-	-
Behavior-1	-	-	-	-	-	0.788
Behavior-2	-	-	-	-	-	0.842
Behavior-3	-	-	-	-	-	0.833

#### Confirmatory Factor Analysis

**Figure 3:**
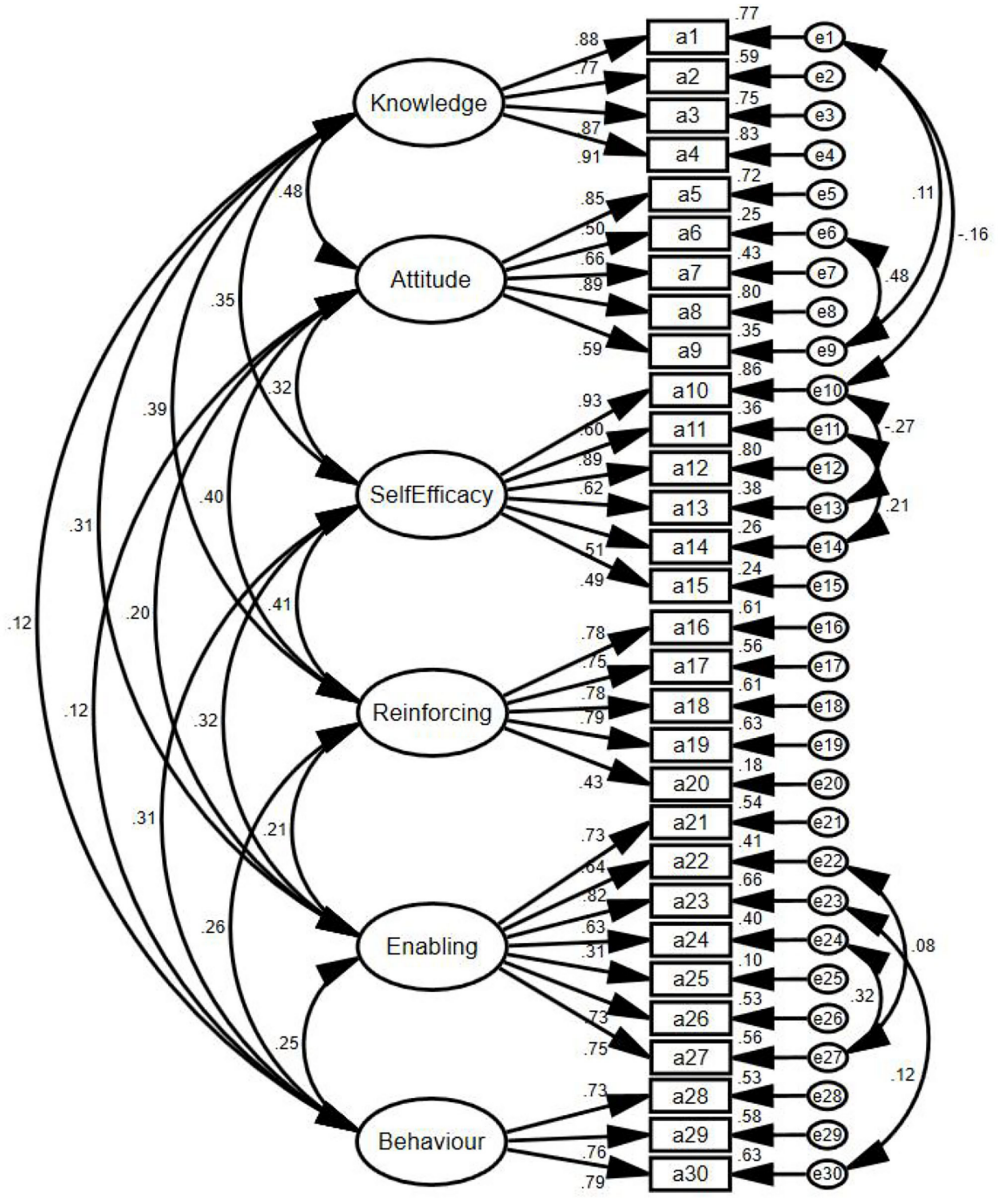
Standardized six-factor structural model of the occupational low back pain prevention behaviors questionnaire.

Based on the 6-factor structure model in AMOS (Figure 3), EFA was completed, and the Maximum Likelihood Estimation (MLE) was adopted. Based on the modification indices (MI), the model was modified 8 times, which were e1 and e9, e1 and e10, e6 and e9, e10, and e13, e11, and e14, e22, and e27, e23, and e30, e24, and e27, respectively. As the results of model fitting, χ^2^/df = 3.753, RMSEA = 0.048, GFI = 0.929, AGFI = 0.913, TLI = 0.934, IFI = 0.943, CFI = 0.943, PGFI = 0.759, PNFI = 0.807.

## Discussion

### The Chinese Version of the Questionnaire Has Suitable Distinction

In this study, we aimed to translate the occupational low back pain prevention behavior questionnaire into Chinese and make cross-cultural adaptation by using the Brislin's double literal translation-back translation model and expert consultation method (29). Seven experts revised the first translation draft according to the actual situation of health promotion among clinical nurses in China and the language expression habits. In the Pre-investigation, 10 clinical nurses also indicated that the Chinese version of the questionnaire had simple and reasonable structure design, clear sentences, easy-to-understand content and strong clinical applicability. The final Chinese version of the occupational low back pain prevention behaviors questionnaire is divided into six dimensions with 30 items in total. The item analysis results also showed that the items of the questionnaire had better discrimination (33), and each item has a medium and high correlation with the whole questionnaire. Cronbach's α value after deleting each item does not exceed the original value of the translated questionnaire. Therefore, the Chinese version of the occupational low back pain prevention behaviors questionnaire has good applicability and discrimination.

### The Chinese Version of the Questionnaire Has Suitable Reliability

In this study, the internal consistency reliability, split-half reliability and test-retest reliability were measured to evaluate the reliability of the Chinese version of the questionnaire. The Cronbach's α coefficient can reflect the homogeneity of items in questionnaire (34). The results showed that the Cronbach's α coefficient of the Chinese version of the questionnaire was 0.885, and the Cronbach's α coefficient of each dimension ranged from 0.903 to 0.924, which is slightly higher than the result of the original questionnaire (24), indicating that the items of the Chinese version of the questionnaire has higher internal consistency. Similarly, the split-half reliability coefficient was 0.663, which once again confirms the above conclusion. The test-retest reliability can reflect the stability and consistency of the test across time (35). The results showed that the test-retest reliability coefficient of the Chinese version of the questionnaire was 0.734, which indicates that the Chinese version of the occupational low back pain prevention behaviors questionnaire has good stability and can be used to measure the occupational low back pain prevention behaviors among clinical nurses.

### The Chinese Version of the Questionnaire Has Suitable Validity

The content validity and construct validity were measured to evaluate the validity of the Chinese version of the questionnaire. In this study, seven experts were invited to evaluate the content validity of the Chinese version of the questionnaire. The results showed that I-CVI ranged from 0.857 to 1.000, and Q-CVI was 0.952, which is higher than the reference value of content validity of 0.9 and 0.8, respectively (36). Furthermore, the EFA results showed that the six common factors of the Chinese version of the questionnaire explained 73.623% of the total variance, and the factor loading of each item in the questionnaire was >0.4, and the factor attribution of each item was consistent with the original version (24), which indicating that the Chinese version of the questionnaire has suitable construct validity. Meanwhile, in CFA, the fitting index of the Chinese version of the questionnaire was within the acceptable range and stronger than that of the original version (24). In conclusion, we consider that the Chinese version of occupational low back pain prevention behaviors questionnaire has suitable content validity and structural validity among clinical nurses.

## Limitation and Perspectives

Some limitations are worth discussing in this study. Although the sample size meets the standard in our research, multi-center large samples are still eager to supplement the existing results. In addition, the questionnaires used in this study are all self-reported, and bias is inevitable. Finally, although we have fully verified the psychometric characteristics of the Chinese version of occupational low back pain prevention behaviors questionnaire among clinical nurses, we have not explored the factors that affect occupational low back pain prevention behavior. Therefore, this is very important for our next work.

## Conclusions

The Occupational Low Back Pain Prevention Behaviors Questionnaire has been successfully introduced into China after translation and cultural adaptation, and its suitable psychometric properties have also been verified among clinical nurses. In response to the high prevalence of occupational low back pain among clinical nurses, the developed questionnaire can provide a reference for nursing managers to formulate education plans and intervention measures to improve clinical nurses' occupational low back pain prevention behaviors.

## Data Availability Statement

The raw data supporting the conclusions of this article will be made available by the authors, without undue reservation.

## Ethics Statement

The studies involving human participants were reviewed and approved by the Ethics Review Committee of Jinzhou Medical University. The patients/participants provided their written informed consent to participate in this study.

## Author Contributions

CZ contributed to data collection, research design, and drafting manuscript. ZY contributed to data collection and data analysis. HZ contributed to manuscript revision, data analysis, and fund management. All authors contributed to the article and approved the submitted version.

## Conflict of Interest

The authors declare that the research was conducted in the absence of any commercial or financial relationships that could be construed as a potential conflict of interest.

## Publisher's Note

All claims expressed in this article are solely those of the authors and do not necessarily represent those of their affiliated organizations, or those of the publisher, the editors and the reviewers. Any product that may be evaluated in this article, or claim that may be made by its manufacturer, is not guaranteed or endorsed by the publisher.
